# Retrieved lymph nodes from different anatomic groups in gastric cancer: a proposed optimal number, comparison with other nodal classification strategies and its impact on prognosis

**DOI:** 10.1186/s40880-019-0394-4

**Published:** 2019-09-13

**Authors:** Siwei Pan, Pengliang Wang, Yanan Xing, Kai Li, Zhenning Wang, Huimian Xu, Zhi Zhu

**Affiliations:** Department of Surgical Oncology, The First Affiliated Hospital of China Medical University, North Nanjing Street 155, Shenyang, 110001 Liaoning P. R. China

**Keywords:** Gastric cancer, American Joint Committee on Cancer, Japanese Gastric Cancer Association, Lymph node, Prognosis, Lymph node ratio, Log odds of metastatic lymph nodes, Stage, Migration, Akaike information criterion, Bayesian information criterion

## Abstract

**Background:**

The optimal number of retrieved lymph nodes (LNs) in gastric cancer (GC) is still debatable and previous studies proposing new classification alternatives mostly focused on the number of retrieved LNs without proper consideration on the anatomic nodal groups’ location. Here, we assessed the impact of retrieved LNs from different nodal location groups on the survival of GC patients.

**Methods:**

Stage I–III gastric cancer patients who had radical gastrectomy were investigated. LN grouping was determined according to the 13th edition of the JCGC. The optimal cut-off values of retrieved LNs in different LN groups (Group 1 and 2) were calculated, based on which a proposed nodal classification (rN) simultaneously accounting the optimal number and location of retrieved LNs was proposed. The performance of rN was then compared to that of LN ratio, log-odds of metastatic LNs (LODDs) and the 8th edition of the Union for International Cancer Control/American Joint Committee on Cancer (UICC/AJCC) N classification.

**Results:**

The optimal cut-off values for Group 1 and 2 were 13 and 9, respectively. The 5-year overall survival (OS) was higher for patients in retrieved Group 1 LNs > 13 (vs. Group 1 LNs ≤ 13, 63.2% vs. 57.9%, *P* = 0.005) and retrieved Group 2 LNs > 9 (vs. Group 2 LNs ≤ 9, 72.5% vs. 60.7%, *P* = 0.009). Patients staged as pN0–3b were sub classified using this Group 1 and 2 nodal analogy. The OS of pN0–N2 patients in retrieved Group 1 LNs > 13 or Group 2 LNs > 9 were superior to those in retrieved Group 1 LNs ≤ 13 and Group 2 LNs ≤ 9 (All *P* < 0.05); except for pN3 patients. The rN classification was formulated and demonstrated better 5-year OS prognostication performance as compared to the LNR, LODDs, and the 8th UICC/AJCC N staging system.

**Conclusions:**

The retrieval of > 13 and > 9 LNs for Group 1 and Group 2, respectively, could represent an alternative lymph node retrieval approach in radical gastrectomy for more precise survival prognostication and minimizing staging migration, especially if > 16 LNs is found to be difficult.

## Background

Gastric cancer (GC) remains the fifth most common malignancy and the third leading cause of cancer-related death in East Asia [1, 2]. Although surgical resection remains the primary curative therapy for GC [3], an accurate staging system is crucial for clinical practice. Before the year 2010 and prior to the wide implementation of classifying lymph nodes (LNs) based on the number of pathologically retrieved LNs, there were two major LN staging systems for GC, namely the Union for International Cancer Control/American Joint Committee on Cancer (UICC/AJCC) classification [4] and the Japanese Gastric Cancer Association (JGCA) staging system [5, 6]. The major difference between the two nodal classifications of GC was that the UICC/AJCC classification was based on the extent of metastatic lymph nodes (mLNs) while the JGCA staging system was based on the anatomic location groups of LNs.

LN metastasis is considered as one of the most important factors affecting the prognosis of GC patients [7–9]. The N stage, based on the number of mLNs, was adopted in the 5th edition of the UICC/AJCC classification, published in 1997 [10]. The JGCA staging system proposed the anatomic nodal classification in the 1st through the 13th editions of the Japanese Classification of Gastric Carcinoma (JCGC), whereas the 14th edition, which was released in 2010, officially abandoned the anatomic nodal classification and adopted a numerical classification similar to that in the UICC/AJCC classification [5, 6]. This shift indicated that the anatomic extent of mLNs was no longer included in the current GC staging system. However, we hypothesized that the potential impact of the anatomic nodal classification for clinical staging and decision making for surgical planning was still worth exploring.

According to the National Comprehensive Cancer Network (NCCN) guidelines, the retrieval of at least 15 LNs is recommended as it is widely accepted that the number of retrieved LNs is closely related to stage migration [11, 12]. Furthermore, many previous studies have indicated that the retrieval of 21 to 23 or even more LNs, partly depending on different TNM stages, should be retrieved to improve prognosis [7, 12–16]. However, most studies did not focus on the anatomic location groups of the retrieved LNs. As indicated in the nationwide Dutch D1D2 trial, the survival superiority of patients who had undergone D2 lymphadenectomy, as compared with D1 lymphadenectomy, was significant, and the retrieval of Group 2 LNs was emphasized [17]. Thus, we speculated that the risk of stage migration could increase when the retrieved LNs were mostly from Group 1 location and it would be more reasonable to determine the number of retrieved LNs according to the anatomic location from a prognostic point of view. If so, perhaps the anatomic location-based nodal categories could show more significance. Additionally, following D2 lymphadenectomy for radical gastrectomy, it is very common that the numbers of retrieved and detected LNs are smaller than the actual number of LNs or LNs-resembling tissues possibly due to the influence of preoperative treatments or lack of efficient communication between the surgeons and pathologists [15, 18, 19]. We hypothesized that an anatomic location-based node category could minimize the impact of the above-mentioned stage migration if an optimal number of LNs were determined and retrieved in different anatomic nodal group locations, not only in Group 1.

Consequently, we analyzed the prognosis of GC patients classified using a proposed investigational anatomic distribution of the involved LNs based on their optimal number of retrieved LNs per location. To determine the clinical implications of this proposed system, its prognostic efficacy was also compared to common lymph node staging systems.

## Patients and methods

### Patient source

**Fig. 1:**
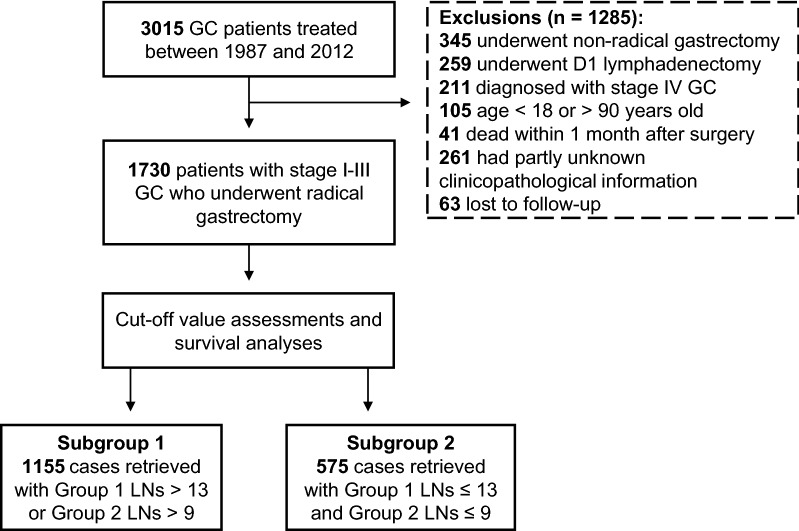
The selection process for stage I–III GC patients enrolled in this study. *GC* gastric cancer, *LNs* lymph nodes. Group 1 and 2 LNs were identified according to the 13th edition of the Japanese Classification of Gastric Carcinoma

The cohort of the current study was limited to patients with stage I–III gastric adenocarcinoma, classified in accordance with the 8th edition of the UICC/AJCC cancer staging manual [4], who underwent radical gastrectomy at the First Hospital of China Medical University (Shenyang, Liaoning, China) between January 1987 and December 2012. Patients with stage IV disease were not included. Patients were excluded based on the following criteria: (1) age < 18 or > 90 years old, (2) the clinicopathological or follow-up information was unknown, and (3) the survival duration after gastrectomy was less than 1 month. Patients who underwent radical gastrectomy combined with D2/D3 lymphadenectomy were selected. The selecting process is shown in Fig. 1.

The following demographics and pathological characteristics were selected for analyses: sex, age, size and site of the primary tumor, histological type, extent of invasion, number of retrieved and metastatic LNs, lymphatic and/or blood vessel invasion (LBVI), follow-up duration, and survival status at last follow-up (November, 2016).

### Surgery procedures

The radical gastrectomy performed in this study cohort included distal, proximal or total gastrectomy, which was mainly determined according to the tumor size, location, and resection margins. Billroth-I, Billroth-II, Roux-en-Y esophagojejunostomy or other types of anastomosis was performed to reconstruct the alimentary tract. The concept of LN grouping, defined as the anatomic location of LNs and tumor, and extent of LN dissection was determined according to the 13th edition of the JCGC (Table 1) [5]. After radical resection, the retrieval of LNs from the resected specimen was performed by one of the surgeons, and the metastatic status of each LN was verified by pathologists.

**Table 1:** Lymph node grouping for gastric cancer patients by anatomic location of tumor according to the 13th edition of the JCGC and clinical practices in our center

LN station	Grouping of the perigastric LNs			
	Overall	Distal	Middle	Upper
No. 1	Group 1	Group 2	Group 1	Group 1
No. 2	Group 1	Distant metastasis	Group 1	Group 1
No. 3	Group 1	Group 1	Group 1	Group 1
No. 4	Group 1	Group 1	Group 1	Group 1
No. 5	Group 1	Group 1	Group 1	Distant metastasis
No. 6	Group 1	Group 1	Group 1	Distant metastasis
No. 7	Group 2	Group 2	Group 2	Group 2
No. 8	Group 2	Group 2	Group 2	Group 2
No. 9	Group 2	Group 2	Group 2	Group 2
No. 10	Group 2	Distant metastasis	Group 2	Group 2
No. 11	Group 2	Group 2	Group 2	Group 2
No. 12	Group 2	Group 2	Group 2	Group 3
No. 13^a^	Group 3	Group 3	Group 3	Distant metastasis

Group 1 and 2 LNs were classified according to the 13th edition of the Japanese Classification of Gastric Carcinoma

*LN* lymph node, *JCGC* Japanese Classification of Gastric Carcinoma

^a^LNs beyond No. 13 station were all defined as Group 3 LNs or distant metastasis

### Follow-up

The follow-up program was based on the NCCN guidelines [11]. In short, patients were followed every 3 months for the first 2 years after gastrectomy, every 6 months for the next 3 years, and annually thereafter. During the follow-up period, the patients received gastroscopy, abdominal computed tomography, or ultrasonography, based on their presenting conditions, and detection of tumor biomarkers to evaluate the surgical outcome and monitor postoperative relapse and metastasis. The endpoint of the current study was overall survival (OS).

### Statistical analysis

Continuous variables were presented as the mean ± standard deviation (SD) and median (interquartile range), while categorical variables were presented as counts and proportions. OS, defined as the time from the date of gastrectomy to death or last follow-up, was calculated using the Kaplan–Meier method and compared using the log-rank test. The cut-off analysis to determine group classification for optimal survival prognostication was used to confirm the most appropriate cut-off values for retrieved LNs in the different groups. X-Tile software (https://medicine.yale.edu/lab/rimm/research/software.aspx) was used to identify the potential cut-off values for each LN group based on minimal probability (*P*) values [20]. Stratification analysis was used to evaluate the influence of different combinations of retrieved LNs.

The LN ratio (LNR) [9] and log-odds of metastatic lymph nodes (LODDs) staging criteria [21] were compared with the staging system proposed in the current study. The LNR stage was identified as the following cut-off values: LNR0: 0%; LNR1: 1–20%; LNR2: 21–50%; LNR3: > 50%. The LODDs stage was classified as follows: LODDs1: LODDs ≤ − 1.5; LODDs2: − 1.5 < LODDs ≤ − 1.0; LODDs3: − 1.0 < LODDs ≤ − 0.5; LODDs4: − 0.5 < LODDs ≤ 0; LODDs5: LODDs > 0 (LODDs = $\log \frac{mLNs + 0.5}{ rLNs - mLNs + 0.5 }$; rLNs referring to the number of retrieved LNs).

The likelihood ratio χ^2^ test was used to assess the homogeneity (no significant differences in survival among patients with the same stage) within each category, and the linear trend χ^2^ test was used to measure the discriminatory ability (significant differences in survival among patients with the different stages) and gradient monotonicity (patients with earlier stages survive longer than those with later stages within the same system). The Akaike information criterion (AIC) and Bayesian information criterion (BIC) values within the Cox proportional hazard regression model were used to evaluate the discriminatory ability of each category. A smaller AIC or BIC value indicated a more desirable model for prediction of OS outcomes [18, 22].

All analyses were conducted using the R software (version 3.4.3; R Foundation for Statistical Computing, Vienna, Austria) and IBM SPSS Statistics for Windows (version 23.0; IBM Corp., Armonk, NY, USA). A two-tailed *P* value < 0.05 was considered statistically significant in all analyses.

## Results

### Clinicopathological characteristics

A total of 1730 patients were found eligible for this study. Their demographics and pathological characteristics are shown in Table 2. The enrolled patients comprised of 1250 males and 480 females, aged 26 to 83 years old, with a median age of 58 (IQR, 50–66 years) years. On average, 23.8 LNs were retrieved from each patient, with more than 15 LNs retrieved from 1177 (68.0%) of the investigated cohort. The median and mean follow-up time was 36.0 and 86.4 months (range, 1 to 426 months), respectively, and no patients were lost to follow-up.

**Table 2:** Demographic and pathological characteristics of the 1730 patients in the current study

Characteristic	All patients	
	*n*	%
Sex		
Male	1250	72.3
Female	480	27.7
Age (years)		
≤ 60	1037	59.9
> 60	693	40.1
Primary tumor site		
Upper	223	12.9
Medium	265	15.3
Lower	1188	68.7
Mixed	54	3.1
Size (cm)		
≤ 4.5	1016	58.7
> 4.5	714	41.3
Median (IQR, cm)	4 (3–6)	
Histological type		
Differentiated	671	38.8
Undifferentiated	1059	61.2
8th UICC/AJCC T stage		
T1	402	23.2
T2	328	19.0
T3	566	32.7
T4a	402	23.2
T4b	32	1.9
8th UICC/AJCC N stage		
N0	812	46.9
N1	331	19.1
N2	294	17.0
N3a	212	12.3
N3b	81	4.7
8th UICC/AJCC TNM stage		
IA	344	19.9
IB	196	11.3
IIA	299	17.3
IIB	283	16.4
IIIA	340	19.7
IIIB	187	10.7
IIIC	81	4.7
LBVI		
Present	353	20.4
Absent	1187	68.6
Unknown	190	11.2
Number of examined LNs		
≤ 15	553	32.0
> 15	1177	68.0
Median (IQR)	21 (13–32)	
Mean ± SD	23.8 ± 14.3	
Number of metastatic LNs		
Median (IQR)	1 (0–4)	
Mean ± SD	3.3 ± 6.0	

*n*, number of patients, *SD* standard deviation, *IQR* interquartile range, *LBVI* lymphatic and/or blood vessel invasion, *LNs* lymph nodes, *UICC/AJCC* Union for International Cancer Control/American Joint Committee on Cancer classification

### Cut-off and survival analyses

**Fig. 2:**
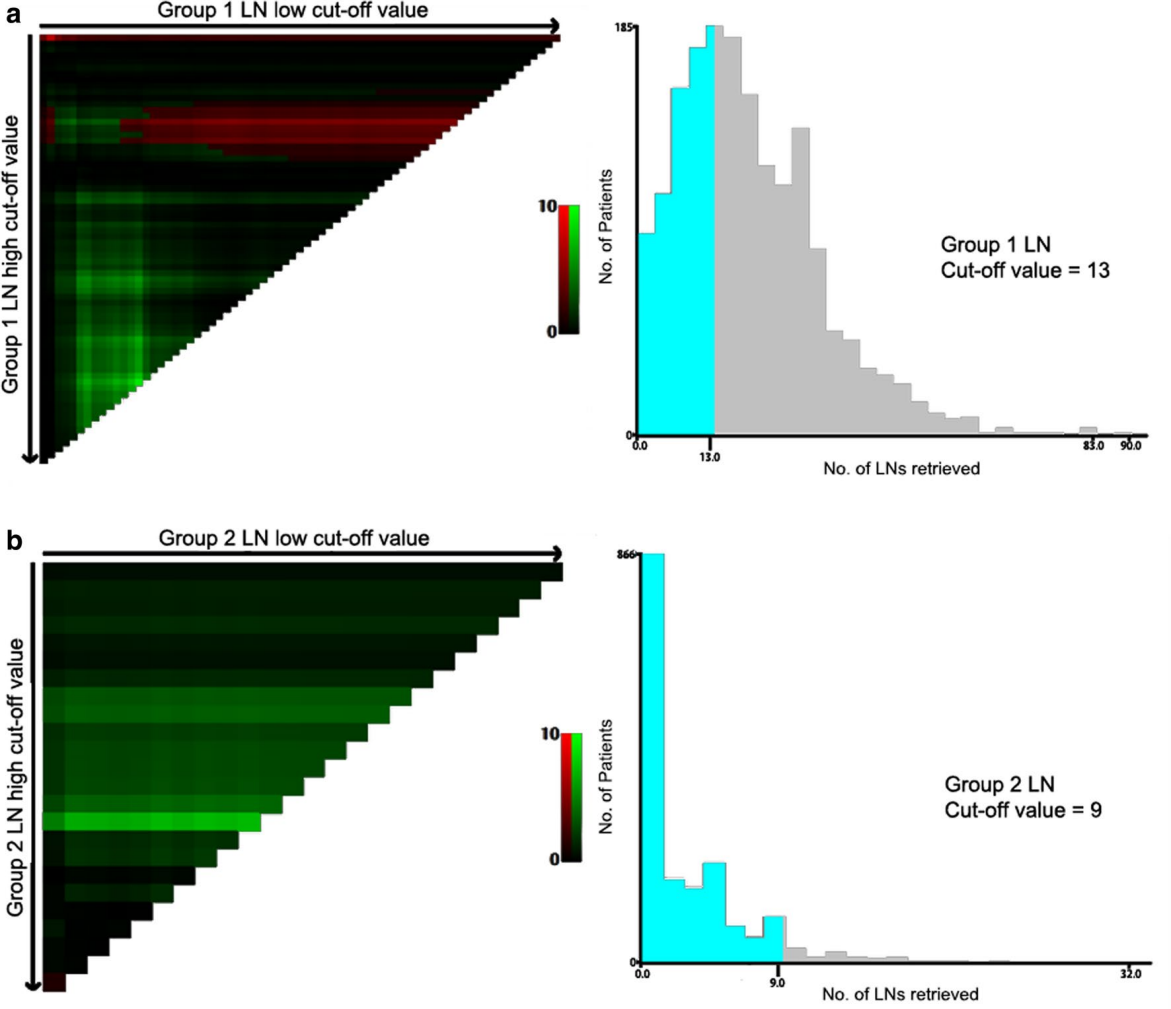
Calculation of the investigated patients using the optimal obtained cut-off values of retrieved Group 1 (**a**) and Group 2 (**b**) LNs using the X-tile software. (The scale refers to χ^2^ log-rank values.) *LNs* lymph nodes

**Fig. 3:**
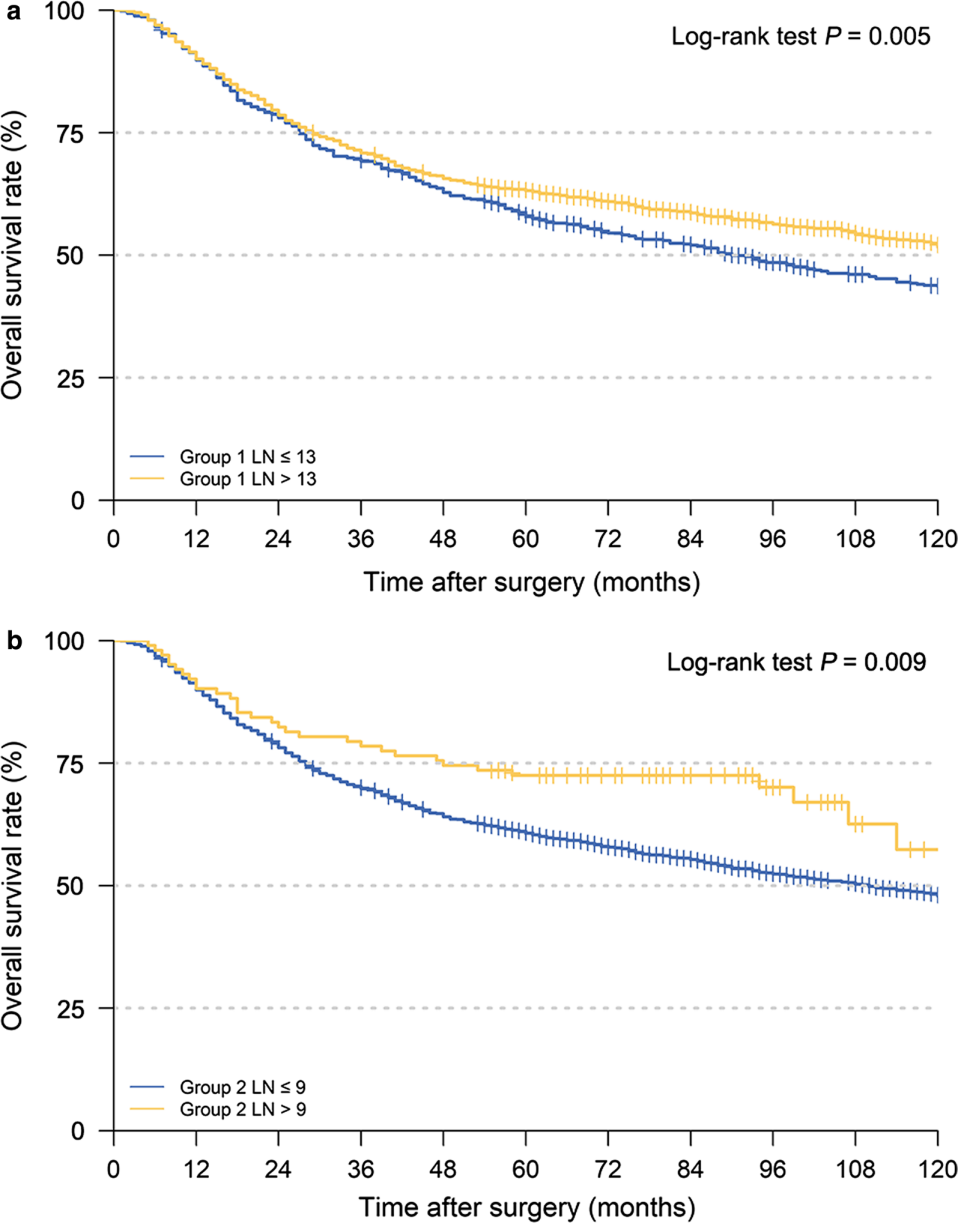
Survival curves of the investigated patients using the optimal obtained cut-off values of retrieved Group 1 (**a**) and Group 2 (**b**) LNs. *LNs* lymph nodes

In the current study, the number of retrieved LNs from Group 1 and Group 2 was assessed. According to the X-tile software, the optimal cut-off values of the numbers of retrieved Group 1 and 2 LNs in this cohort were 13 and 9, respectively (Fig. 2). Survival analyses indicated that the 5-year OS was higher for patients in retrieved Group 1 LNs > 13, as compared to those in retrieved Group 1 LNs ≤ 13 (63.2% vs. 57.9%, respectively, *P* = 0.005; Fig. 3a). Similarly, the retrieval of LNs for patients in Group 2 LNs > 9 was also associated with a better prognosis than those in Group 2 LNs ≤ 9 (5-year OS, 72.5% vs. 60.7%, respectively, *P* = 0.009; Fig. 3b).

**Fig. 4:**
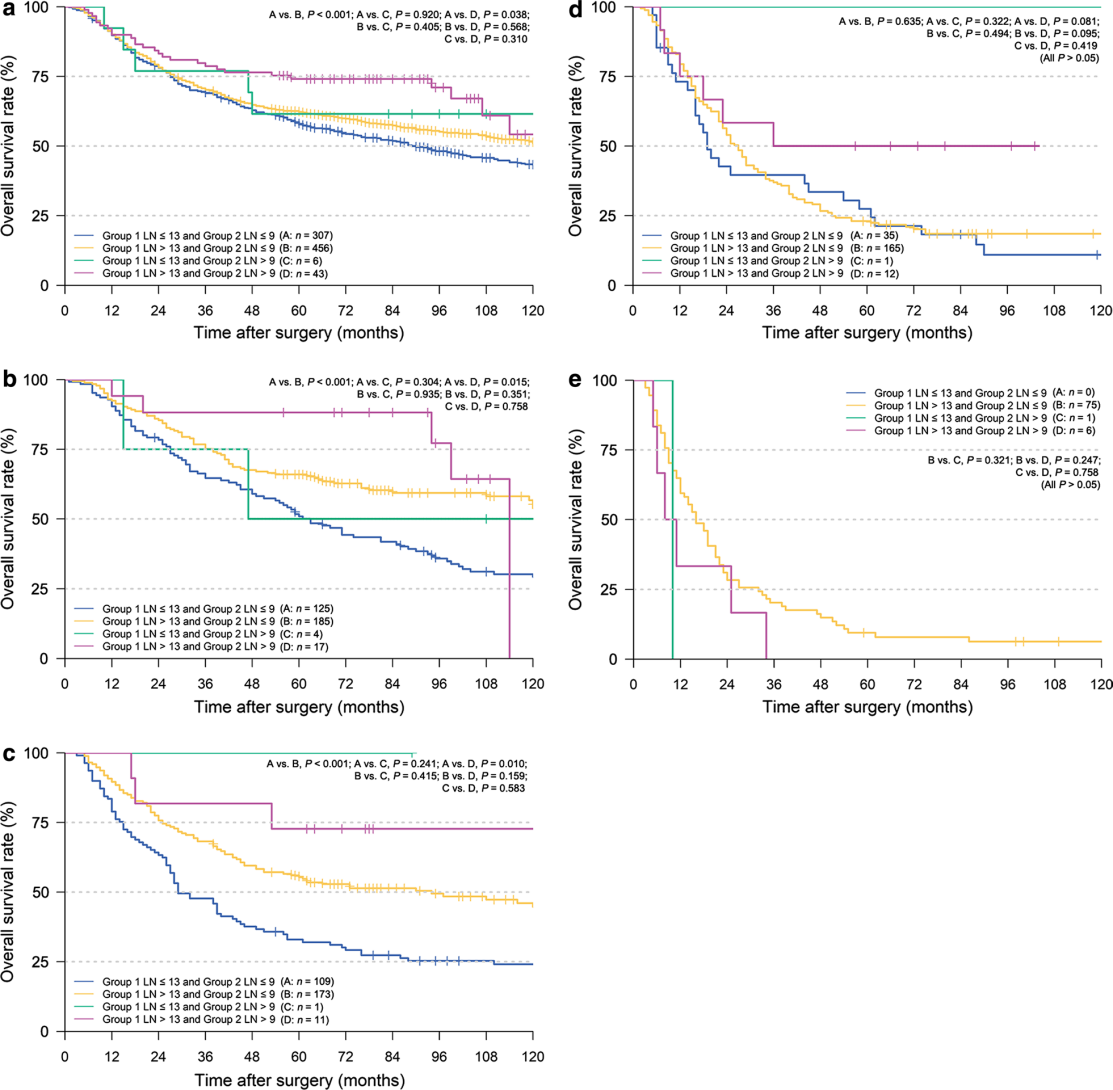
Kaplan–Meier OS curves of gastric cancer patients at different N stages stratified according to the number of retrieved LNs from different locations. **a** pN0 stage; **b** pN1 stage; **c** pN2 stage; **d** pN3a stage; **e** pN3b stage. *OS* overall survival, *LNs* lymph nodes, *pN* pathological lymph node classification

The OS curves of patients staged as pN0–pN3, stratified based on their number of retrieved LNs from different locations (Group 1 and 2), are illustrated in Fig. 4. Different nodal grouping combinations using the cut-off values 13 and 9 for retrieved Group 1 and 2 LNs, labeled as A, B, C, and D, were investigated. The OS of patients classified as Group 1 LN > 13 or Group 2 > 9 were significantly better than those classified as Group 1 LN ≤ 13 and Group 2 ≤ 9 (Fig. 4a–c), except for nodal stage pN3 (Fig. 4d, e) which may have been caused due to the limited number of patients in stage pN3.

**Fig. 5:**
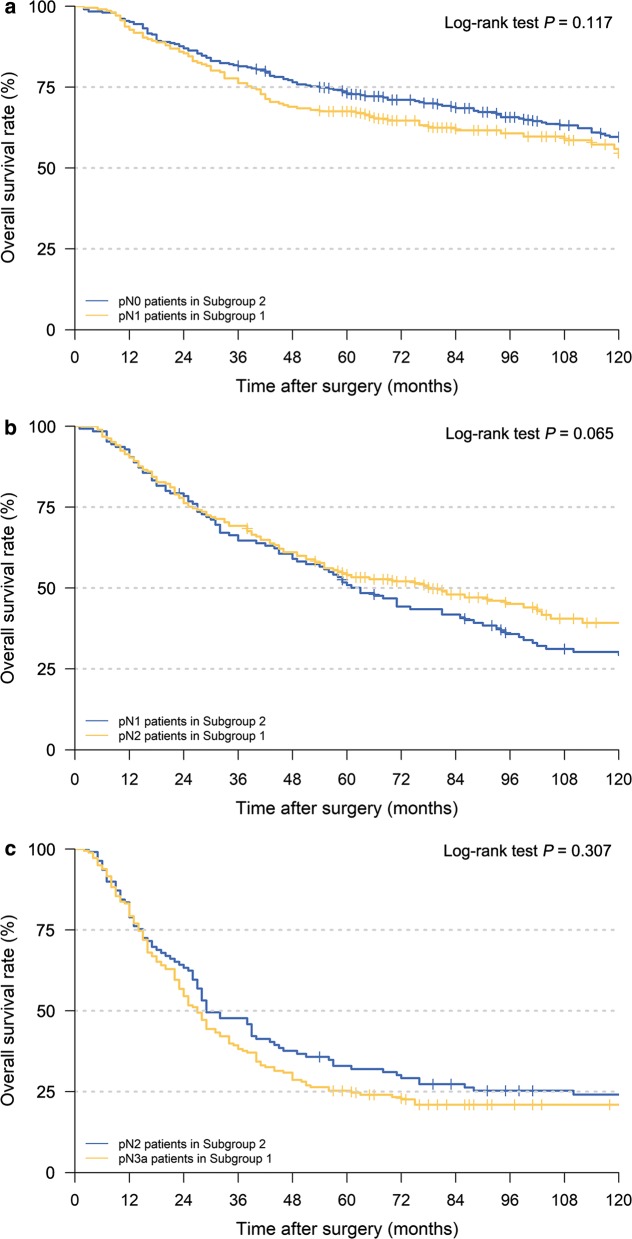
Kaplan–Meier OS curves of patients under different pN stages assigned to different subgroups. **a** pN0 patients in Subgroup 2 vs. pN1 patients in Subgroup 1, **b** pN1 patients in Subgroup 2 vs. pN2 patients in Subgroup 1, **c** pN2 patients in Subgroup 2 vs. pN3a patients in Subgroup 1. (Subgroup 1: patients with retrieval of Group 1 LNs > 13 or Group 2 LNs > 9; Subgroup 2: patients with retrieval of Group 1 LNs ≤ 13 and Group 2 LNs ≤ 9). *OS* overall survival,* pN* pathological lymph node classification

The entire cohort was then divided into two groups according to the most appropriate cut-off values in the two LN groups: patients with the retrieval of Group 1 LNs > 13 or Group 2 LNs > 9 (Subgroup 1) vs. retrieval of Group 1 LNs ≤ 13 and Group 2 LNs ≤ 9 (Subgroup 2). No significant differences in OS were observed between pN0 stage patients in Subgroup 2 and pN1 stage patients in Subgroup 1 (Fig. 5a, *P* = 0.117), between pN1 stage patients in Subgroup 2 and pN2 stage patients in Subgroup 1, as well as between pN2 stage patients in Subgroup 2 and pN3a stage patients in Subgroup 1 (Fig. 5b, c; all *P* > 0.05).

Based on the cut-off values of different LN groups and survival analyses, we proposed a revision to the N staging of the 8th edition of the UICC/AJCC GC classification (Table 3) [4] (i.e., the revised N1 category also contained the UICC/AJCC-pN0 stage patients in Subgroup 2 and UICC/AJCC-pN1 stage patients in Subgroup 1).

**Table 3:** rN staging for gastric cancer patients after radical gastrectomy according to the numbers of retrieved Group 1 and 2 LNs

8th UICC/AJCC N stage/(no. of patients)	Examined LNs/(no. of patients)	
	Group 1 > 13 or Group 2 > 9 (Subgroup 1)	Group 1 ≤ 13 and Group 2 ≤ 9 (Subgroup 2)
N0 (*n* = 812)	rN0 (*n* = 505)	rN1 (*n* = 307)
N1 (*n* = 331)	rN1 (*n* = 206)	rN2 (*n* = 125)
N2 (*n* = 294)	rN2 (*n* = 185)	rN3 (*n* = 109)
N3a (*n* = 212)	rN3 (*n* = 178)	rN3 (*n* = 34)
N3b (*n* = 81)	rN4 (*n* = 81)	rN4 (*n* = 0)

*N* nodal stage based on the 8th edition of the Union for International Cancer Control/American Joint Committee on Cancer classification (UICC/AJCC) classification, *rN* revised nodal stage

### Comparison of the accuracies of the different staging systems in prognostic prediction

**Fig. 6:**
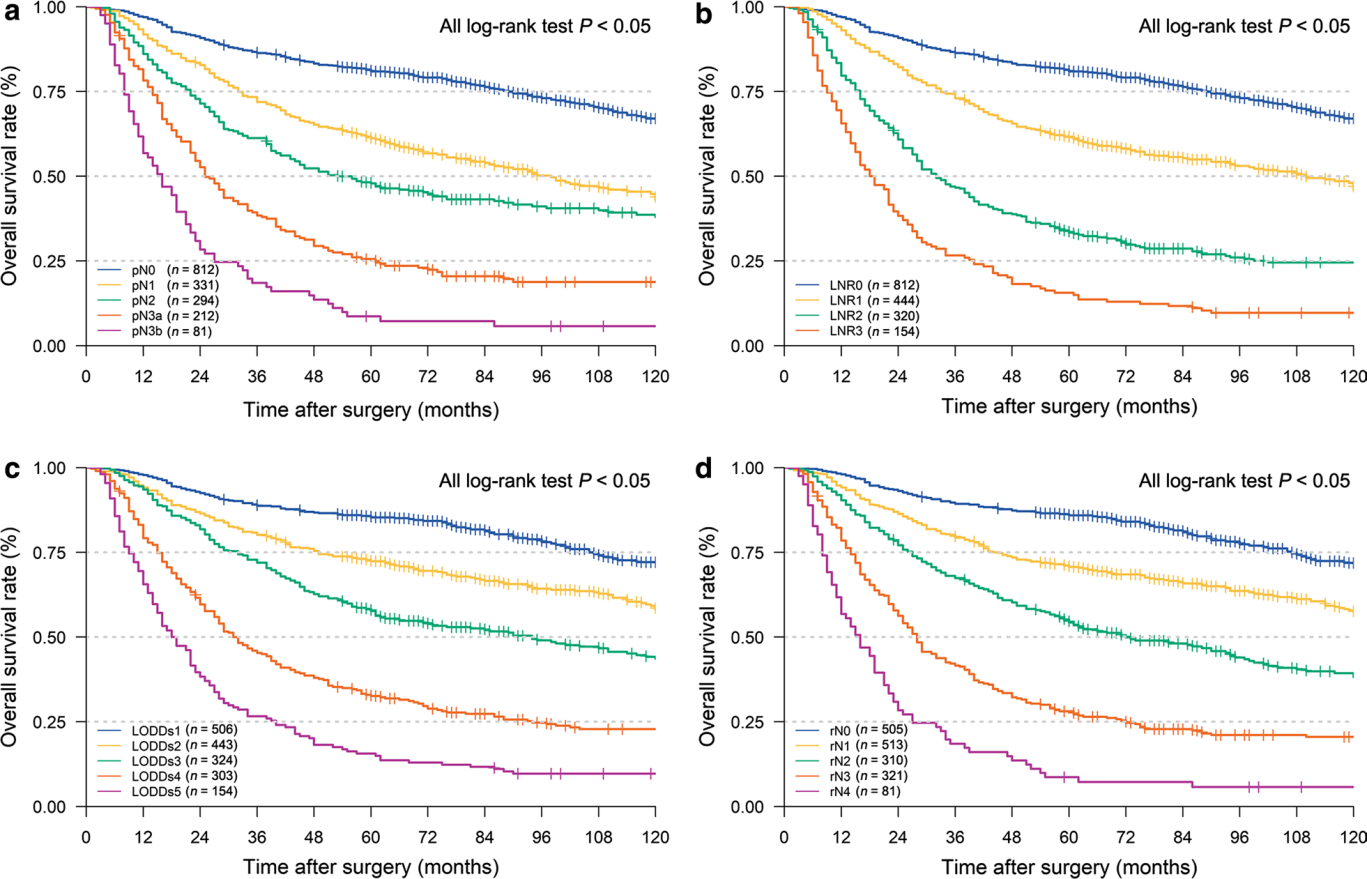
Kaplan–Meier OS curves of gastric cancer patients with nodal statuses classified according to different staging systems. **a** UICC/AJCC-pN staging, **b** LNR staging, **c** LODDs staging, **d** revised N staging. *pN* pathological lymph node classification based on the 8th edition of the Union for International Cancer Control/American Joint Committee on Cancer classification, *LNR* lymph node ratio, *LODDs* log odds of metastatic lymph nodes, *rN* revised lymph nodal stage

The OS curves, according to the 8th edition of the UICC/AJCC-N, LNR [9], LODDs [21] and the revised stage are illustrated in Fig. 6. The homogeneity, discriminatory ability, and monotonicity of gradients were improved with the revised N staging system, with higher liner trend χ^2^ and likelihood ratio χ^2^ values than the UICC/AJCC-N staging, LNR stage and LODDs stage (Table 4). Lastly, the AIC and BIC of our revised N staging system were smallest among the four systems investigated nodal staging systems, suggesting that the revised N staging system might be an optimal prognosis stratification system.

**Table 4:** Comparison of the performance of the AJCC-N, LNR, LODDs, and the rN staging systems in predicting prognosis of gastric cancer

Staging system	Liner trend χ^2^	Likelihood ratio χ^2^	AIC	BIC
UICC/AJCC-N	201.522 (*P* < 0.001)*	360.823 (*P* < 0.001)^*#*^	12,830.84	12,836.29
LNR	232.258 (*P* < 0.001)*	365.131 (*P* < 0.001)^*#*^	12,826.56	12,831.44
LODDs	294.031 (*P* < 0.001)*	390.501 (*P* < 0.001)^*#*^	12,803.69	12,808.58
rN	313.340 (*P* < 0.001)*	410.575 (*P* < 0.001)^*#*^	12,785.33	12,790.78

*AIC* Akaike information criterion, *BIC* Bayesian information criterion, *N* nodal stage based on the 8th edition of the Union for International Cancer Control/American Joint Committee on Cancer classification (UICC/AJCC), *LNR* lymph node ratio, *LODDs* log odds of metastatic lymph nodes, *rN* revised nodal stage

* Comparison of overall survival by liner trend χ^2^ test among different stages

^#^Comparison of overall survival by likelihood ratio χ^2^ test among different stages

## Discussion

In the current study, we analyzed the prognostic impact of the different number of retrieved LNs in Group 1 and 2 LNs on 1730 GC patients who underwent radical gastrectomy. We found that the optimal number of retrieved LNs for Group 1 and 2, based on the 13th edition of the JCGC, was > 13 and 9 respectively, based on which we proposed a revised nodal classification (rN). The prognostic prediction of patients classified using the rN criteria was found to be superior than those classified using the 8th UICC/AJCC GC, LNR, and LODDs criteria.

The number of retrieved LNs serves as a prognostic factor for GC as well as for postoperative survival for certain cancers [23]. The NCCN and TNM staging guidelines recommend the resection of no less than 15 LNs for radical gastrectomy [11, 24, 25]. However, the optimal number of retrieved LNs remains controversial. For example, Hayashi et al. [13] recommended retrieval of > 40 LNs after total gastrectomy for stage III patients, whereas Lu et al. [12] suggested that harvesting 21 LNs might represent a superior cut-off point for radical total gastrectomy to better determine the prognosis of the patients. Although a greater number of retrieved LNs has been associated with longer survival of patients with node-metastatic cancer, the optimal number of LNs to be examined at different stages remains unclear.

Nevertheless, almost all previous studies have focused exclusively on the number of retrieved LNs, both metastatic and non-metastatic, without proper consideration on their residing anatomic location groups [18, 26]. It has been indicated in esophageal cancer that both the location and number of metastatic LNs had important prognostic impact [27]. In GC, Zhao et al. [28] reported that the anatomical location of metastatic LNs was an important prognostic factor, especially in patients with stage pN1–N2 disease, whereas Tong et al. [29] emphasized that the classification of LNs in different locations did affect treatment and prognostic assessment. Although it is widely accepted that stage migration is related to the prognosis and the number of retrieved LNs, stage migration and survival prognostication discrepancies can occur when LNs are mostly removed from stations 1–7 as compared to other stations, based on previous studies [17, 27–29] and our own clinical experience. Moreover, skip metastasis of LNs or solitary metastatic LNs in GC is common, and it is necessary to retrieve Group 2 LNs to increase the probability of removing as much mLNs as possible [30, 31]. Thus, whether the number-based nodal category is superior to the revised anatomic location-based nodal category deemed worthy of investigation, and the current study intended to combine both methodologies.

Our findings showed significant associations between the number of retrieved LNs at different anatomic location groups and outcomes of patients with GC. On average, 23.8 LNs were retrieved from each patient, with more than 15 retrieved from 1177 (68.0%) of the cohort. X-tile software was employed to calculate the cut-off values of retrieved Group 1 and Group 2 LNs in predicting survival outcomes. We found that the retrieval of > 13 and > 9 LNs for Group 1 and Group 2 was associated with a relatively better prognosis. Additionally, combinations of different numbers of retrieved Group 1 and Group 2 LNs was confirmed to have different effects on the prognosis of patients classified by pN stage, as their prognosis was significantly poorer for Subgroup 2 patients than for Subgroup 1 patients, especially among those with stage pN0–N2 disease. Nevertheless, for pN3 patients, there was no significant difference observed in the prognosis between Subgroup 1 and 2. This may have been because patients needed to have a range of 7–15 and > 16 LNs retrieved to be classified as pN3a and pN3b, respectively, which mostly englobed our proposed criteria Group 1 > 13 LNs and 2 > 9 LNs; thereby possibly reflecting no significant difference with that of our proposed grouping criteria. Hence, based on the findings presented, we suggest that the importance of anatomic location groups of the retrieved LNs should not be ignored.

Notably, D2 lymphadenectomy has been accepted as an important part of radical gastrectomy and standard treatment to manage LN metastasis [5, 17, 32, 33]. However, the number of retrieved LNs is influenced by the extent of lymphadenectomy, surgical choice, the surgeon’s skill and/or the ability to examine LNs by the surgeons or pathologists. Sometimes, LNs are retrieved by pathologists who are less familiar with the anatomic locations. Therefore, it is inevitable that the number of LNs retrieved from various stations will differ among surgeons, centers, and even countries. Furthermore, it is very difficult to retrieve a sufficient number of LNs in some patients after neoadjuvant chemoradiotherapy. Under such circumstances, the examination of insufficient LNs could result in stage migration and affect the prognostic evaluation and the formulation of the optimal treatment to be given. Consequently, it is necessary and reasonable to formulate a method able to agglomerate both the number and distribution of retrieved LNs. To this end, we integrated our results into the UICC/AJCC-N staging system. In the proposed revision of the N staging system, for example, stage rN0 was limited to patients with UICC/AJCC-pN0 stage in Subgroup 1 (Table 3), whereas the N1 stage rN1 contained patients with UICC/AJCC-pN0 stage in Subgroup 2 and UICC/AJCC-pN1 stage in Subgroup 1 on the account that there was no significant prognostic difference between these two subgroups. Furthermore, after comparing LNR with LODDs staging systems, the revised N staging system demonstrated superior prognostic stratification and was found to focus more on the retrieved number of LNs in Group 1 and 2. We thought the superiority might be that we rationalized the sources of retrieved LNs and distinguished different combinations of retrieved LNs. Further, implementation of this proposed system, if properly validated, may be easier as compared to the LNR or LODDs which requires some level of mathematical calculation before use. The findings from the present study not only showed that the revised N staging system was superior and clinically feasible, but also suggest that gastric cancer surgeons should be paying more attention to the anatomic locations of the retrieved LNs as this can enable surgeons to increase the number of retrieved LNs and improve the survival prognostication of their patients.

There were several limitations in the present study that should be addressed. First, the revised N staging system was based on the analyses of data from a single institution in China, thus the results regarding the number of harvested LNs may differ among institutions. Second, our conclusions were based on data collected between 1987 and 2012 and we considered that this was a relatively long period that may have caused heterogeneity in diagnosis, treatment skills, and postoperative treatment recommendations. Third, the current study only focused on Group 1 and 2 LNs, thus it might be more precise for the N staging system to include the numbers of LNs retrieved from all LN groups mentioned in the JCGC [5]. Furthermore, it was inevitable that for patients who underwent total gastrectomy, it was easier to retrieve more than 13 LNs in the Group 1 LNs as compared to other gastric resection types. These above-mentioned limitations could have caused the deviation of patient classification and affect the conclusions of this study, to a certain extent. Hence, a prospective study recruiting more patients to validate the conclusions from the current study is to be considered in the future.

## Conclusions

The results of the current study indicated that the retrieval of > 13 LNs for Group 1 or > 9 LNs for Group 2 LNs can lead to better survival outcomes for patients with stage I–III pN0–N2 GC. The revised N staging system showed improved homogeneity, discriminatory ability, and monotonicity of gradients than AJCC-N, LNR, and LODDs staging system. Our study also suggested that this revised system can be used to minimize stage migration if the retrieval of > 16 LNs is found to be difficult.

## Data Availability

The datasets used and/or analyzed during the current study are available from the corresponding author on reasonable request.

## Abbreviations

GC: gastric cancer
UICC/AJCC: Union for International Cancer Control/American Joint Committee on Cancer
JGCA: Japanese Gastric Cancer Association
LN: lymph node
mLNs: metastatic lymph nodes
NCCN: National Comprehensive Cancer Network
LBVI: lymphatic and/or blood vessel invasion
TNM: tumor, node and metastasis
SD: standard deviation
JCGC: Japanese Classification of Gastric Carcinoma
OS: overall survival
AIC: Akaike information criterion
BIC: Bayesian information criterion
